# Assessing models of speciation under different biogeographic scenarios; an empirical study using multi‐locus and RNA‐seq analyses

**DOI:** 10.1002/ece3.1865

**Published:** 2016-01-07

**Authors:** Taylor Edwards, Marc Tollis, PingHsun Hsieh, Ryan N. Gutenkunst, Zhen Liu, Kenro Kusumi, Melanie Culver, Robert W. Murphy

**Affiliations:** ^1^ School of Natural Resources and the EnvironmentThe University of ArizonaTucsonArizona85721; ^2^ University of Arizona Genetics CoreUniversity of ArizonaTucsonArizona85721; ^3^ School of Life SciencesArizona State UniversityTempeArizona85287; ^4^ Department of Ecology and Evolutionary BiologyThe University of ArizonaTucsonArizona85721; ^5^ Department of Molecular and Cellular BiologyThe University of ArizonaTucsonArizona85721; ^6^ State Key Laboratory of Genetic Resources and EvolutionKunming Institute of ZoologyChinese Academy of SciencesKunming650223China; ^7^ Arizona Cooperative Fish & Wildlife Research UnitUSGSUniversity of ArizonaTucsonArizona85721; ^8^ Centre for Biodiversity and Conservation BiologyRoyal Ontario MuseumTorontoONCanada

**Keywords:** ∂a∂i, allopatric, gene flow, *Gopherus*, parapatric, phylogenetic, transcriptome

## Abstract

Evolutionary biology often seeks to decipher the drivers of speciation, and much debate persists over the relative importance of isolation and gene flow in the formation of new species. Genetic studies of closely related species can assess if gene flow was present during speciation, because signatures of past introgression often persist in the genome. We test hypotheses on which mechanisms of speciation drove diversity among three distinct lineages of desert tortoise in the genus *Gopherus*. These lineages offer a powerful system to study speciation, because different biogeographic patterns (physical vs. ecological segregation) are observed at opposing ends of their distributions. We use 82 samples collected from 38 sites, representing the entire species' distribution and generate sequence data for mtDNA and four nuclear loci. A multilocus phylogenetic analysis in *BEAST estimates the species tree. RNA‐seq data yield 20,126 synonymous variants from 7665 contigs from two individuals of each of the three lineages. Analyses of these data using the demographic inference package ∂a∂i serve to test the null hypothesis of no gene flow during divergence. The best‐fit demographic model for the three taxa is concordant with the *BEAST species tree, and the ∂a∂i analysis does not indicate gene flow among any of the three lineages during their divergence. These analyses suggest that divergence among the lineages occurred in the absence of gene flow and in this scenario the genetic signature of ecological isolation (parapatric model) cannot be differentiated from geographic isolation (allopatric model).

## Introduction

Geography, gene flow, and time strongly influence speciation, but the relative importance of these mechanisms can be difficult to quantify (Via 2009; Pinho and Hey 2010). Competing species concepts often differ fundamentally in the contribution of gene flow to the process of speciation. Recently diverged taxa facilitate studying the influence of gene flow on speciation because the signature of past introgression may persist in the genome (Pinho and Hey 2010).

It is difficult to test empirically for signatures of past introgression in natural systems due to pervasive genetic drift, genetic draft and variation in coalescence times (Hudson and Turelli 2003). Differences among gene genealogies may arise from differences in male/female dispersal, assortative mating and differential selection (Coyne and Orr 2004). Such processes result in discordance among gene trees of recently diverged species (Pollard et al. 2006; Degnan and Rosenberg 2009; Zhang 2011) and it is difficult to discriminate patterns of lineage sorting from patterns of past introgression because they have similar genetic signatures (McCormack et al. 2009; Payseur 2010; Pinho and Hey 2010). The competing explanations of discordance between gene trees render the study of the contribution of gene flow to speciation in natural systems challenging, although the more loci examined throughout the genome, the more likely a clear phylogenetic signal can be distinguished (Leaché and Rannala 2011).

Recent advances in biotechnology and biostatistics enable investigations into speciation. New molecular technologies and multi‐locus genomic methods can fuse evolutionary history within an ecological context (Brito and Edwards 2009). Genomic approaches also allow for simultaneous exploration of differences in introgression among different parts of the genome (Payseur et al. 2004; Geraldes et al. 2006; Teeter et al. 2008; Melo‐Ferreira et al. 2009). This integration of population genetic and phylogenetic perspectives promotes the creation of meaningful species trees (Degnan and Rosenberg 2009).

Explorations into the relative importance of divergence and gene flow require identifiable patterns of speciation, such as cases in which two recently diverged populations come into secondary contact. Ecotones between two distinct habitats facilitate the testing of hypotheses on patterns of divergence. In this situation, hybridization may occur and it can be an important part of the evolutionary process and an essential component in species' ability to adapt to a changing environment (Barton and Hewitt 1989; Arnold 2007; Payseur 2010).

**Figure 1:**
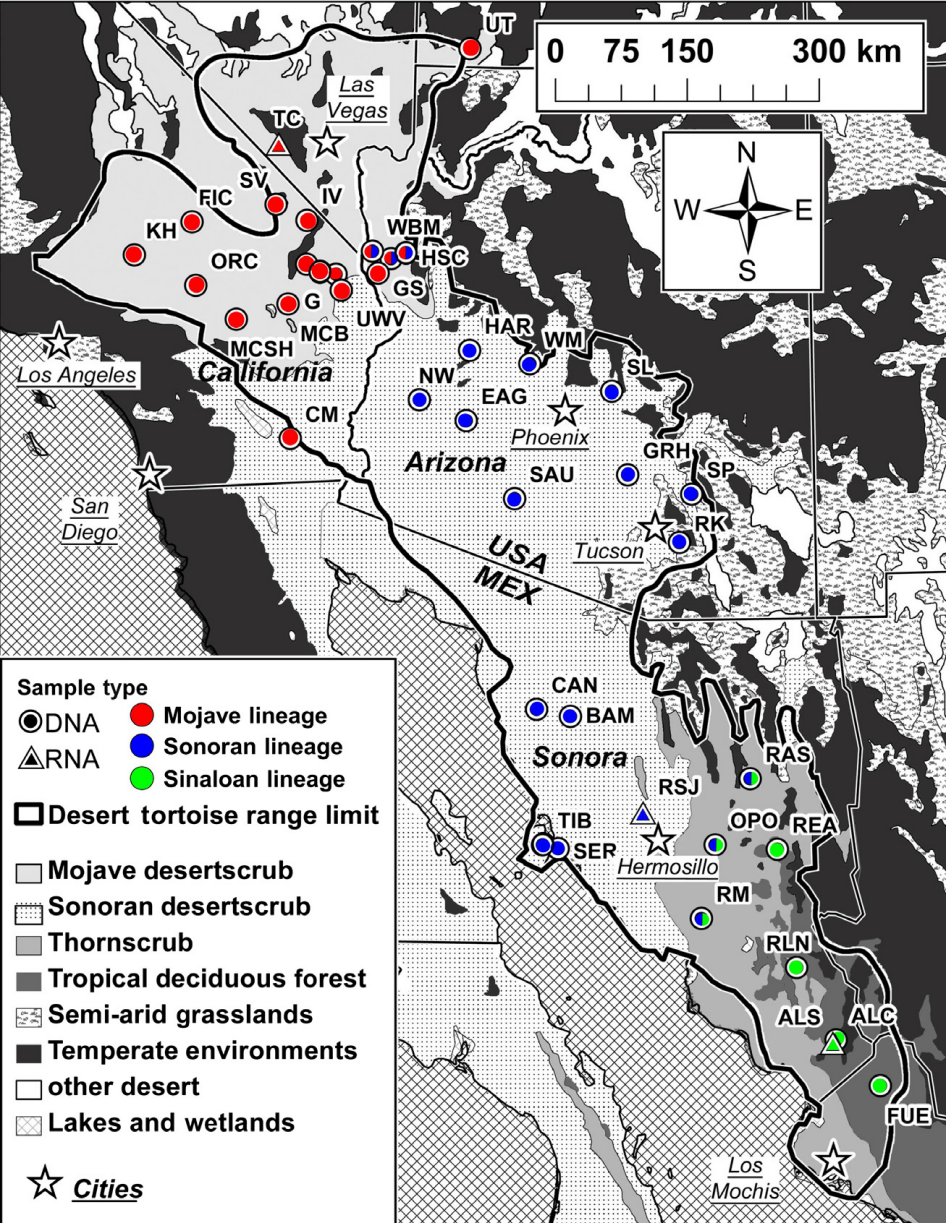
Map of desert tortoise sampling locations where Site IDs correspond with Table 1. DNA samples obtained from locations marked with a circle; RNA samples obtained from sites marked with a triangle. Hybrid zones where lineages come into contact represented by circles with split colors. Habitat distribution estimated from (Brown and Lowe 1980) and by digitizing published maps in Búrquez et al. (1999) and Felger et al. (2012). Desert tortoise range limit from Germano et al. (1994).

Desert tortoises (*Gopherus* sp.) lend themselves well to testing for the drivers of speciation and the roles played by ecology because they are recently diverged and wide‐ranging in multiple biomes (Fig. 1). Phylogenetic reconstruction of mtDNA haplotypes suggest a trichotomy of similarly, divergent matrilines that strongly associate with geography (Edwards et al. 2012, 2015b). Previous estimates of mtDNA divergence time between lineages of desert tortoise have been fairly consistent at 5–6 Ma (Avise et al. 1992; Lamb and Lydeard 1994; McLuckie et al. 1999; Edwards 2003). Importantly, regions of overlap occur between the distributions of divergent lineages. At these sites, hybridization is ongoing and there may be signals of past introgression (McLuckie et al. 1999; Edwards et al. 2010). Ecotones define the distribution of divergent lineages and selection appears to maintain taxonomic boundaries where they come into contact (Edwards et al. 2015a, 2015b). Furthermore, the three lineages in this system allow obtainment of a consensus among multiple gene genealogies, as there is greater potential to converge on an incorrect species tree when four or more taxa exhibit discordance among gene trees (Degnan and Rosenberg 2009).

*Gopherus agassizii* (Agassiz's desert tortoise) and *G. morafkai* (Morafka's desert tortoise) are a classic example of allopatric speciation resulting from geographic isolation by the Colorado River (Lamb et al. 1989; Avise et al. 1992; McLuckie et al. 1999; Murphy et al. 2011). The former species occurs primarily west and north of the Colorado River in the Mojave Desert and *G. morafkai* ranges solely south and east of the river in the Sonoran Desert. A small population of *G. agassizii* occurs on the east side of the Colorado River in the territory of *G. morafkai* (McLuckie et al. 1999). Edwards et al. (2015a) used microsatellite and mtDNA genotypic data and performed habitat suitability modeling to characterize this secondary contact zone in northwestern Arizona. The distribution of each species strongly correlated with topographic, climatic, and vegetative variables. A relatively small number of hybrid individuals, most of which were identified as *F*_2_ or backcrossed individuals, lived in ecotonal areas only. A limited distance of penetration from either parental or hybrid genotype class occurred across the contact zone. Ecological niche partitioning apparently maintains the species via a geographical selection‐gradient.

In the southern part of the range of *G. morafkai*, parapatry may explain the formation of genetically and geographically distinct “Sonoran” and “Sinaloan” lineages (Edwards et al. 2012). The Sonoran genotype has a large distribution throughout desertscrub in Sonora, Mexico, and Arizona, USA. In contrast, the southern, Sinaloan lineage occurs solely in tropical deciduous forest and thornscrub environments (Fig. 1; Edwards et al. 2015b). The lineages occur sympatrically in a narrow ecotone between the two habitats and limited hybridization has been detected (Edwards et al. 2015b). No geographic barrier limits gene flow. Although this pattern implicates a parapatric model of speciation, these desert and tropical environments likely expanded and contracted many times during the Pleistocene (Van Devender 2000; Riddle and Hafner 2006); this dynamic system has undoubtedly influenced speciation of the biota.

Edwards et al. (2015b) performed a clinal analysis of the zone of overlap between the Sonoran and Sinaloan lineages using microsatellite and mtDNA data. A bimodal distribution of genotypes with a strong coincidence of slope and concordance of center between clines supported the hypothesis of secondary contact (Endler 1977; Barton and Hewitt 1985). The current contact zone between the Sonoran and Sinaloan lineages appeared to result from secondary contact after periods of isolation in Pleistocene refugia. Hybrid zones between parapatric taxa typified repeated population contractions into refugia followed by expansions during climate oscillations (Hewitt 1996). Edwards et al. (2015b) suggested that the shifting ecotone between tropical deciduous forest and Sonoran desertscrub likely acted as an ephemeral boundary providing recurring opportunities for interbreeding, which may have reinforced niche segregation in each lineage of tortoise. They characterized the Sonoran and Sinaloan lineages of *G. morafkai* as having independent evolutionary trajectories despite incomplete reproductive isolation.

The underlying population structure of an organism is critical to making inferences about the rate and patterns of speciation. Within each of the *G. agassizii* (hereto referred to as the “Mojave” lineage) and the *G. morafkai* Sonoran and Sinaloan lineages, gene flow is geographically extensive and there is genetic structure with isolation by distance (IBD: Edwards et al. 2004; Murphy et al. 2007; Hagerty et al. 2011; Edwards et al. 2015a, 2015b). All three lineages appear to have experienced population expansions since the Last Glacial Maximum (LGM) based on mtDNA analysis; star phylogenies are observed at the tips of each of the long, matrilineal branches (Edwards 2003; Edwards et al. 2015b). Populations within each lineage have low estimates of genetic differentiation: *F*_ST_ = 0.06 for *G. agassizii*;* F*_ST_ = 0.05 for *G. morafkai* Sonoran; and *F*_ST_ = 0.09 for *G. morafkai* Sinaloan (Edwards and Harrison 2014; T. Edwards unpubl. data). This suggests that any sampling location within a lineage should contain roughly 90–95% of the genetic diversity of that lineage. At contact zones between lineages, if no species boundary occurs, then gene introgression should follow an IBD model. In contrast, where taxonomic boundaries are maintained at contact zones, then the rate of introgression for alleles that are under strong selection should be near zero in one or both directions (Payseur 2010).

Herein, we test hypotheses regarding biogeography and its influence on drivers of speciation. These involve comparisons of the well‐established allopatric model of speciation observed between the Sonoran/Mojave lineages with predictions of the parapatric model between Sonoran/Sinaloan lineages. Under a parapatric model of speciation we expect that a signature of past introgression may persist in the genome because divergence occurred with the potential for gene flow, whereas under an allopatric model there would be no opportunity for introgression during divergence. The crux of this study system is that within this trichotomy of recently diverged taxa, the Mojave/Sonoran allopatric model provides a base line for comparison with the Sonoran/Sinaloan parapatric model. This helps reduce the background noise caused by incomplete lineage sorting. Because hybridization currently occurs among the lineages, it would be assumed that any past events might leave a genetic signature. For the analyses, we use mitochondrial DNA (mtDNA), nuclear loci (nDNA), and RNA‐seq data.

### Hypotheses

We test hypotheses that the three lineages of desert tortoises experienced different mechanisms of speciation. Hypothesis H‐MS_i_ (Mojave/Sonoran isolation) assumes that the Mojave and Sonoran lineages experienced divergence in isolation. Alternatively, H‐MS_gf_ (Mojave/Sonoran gene flow) involves divergence with gene flow. Hypothesis H‐SS_gf_ (Sonoran/Sinaloan gene flow) assumes that the Sonoran and Sinaloan lineages experienced parapatric speciation without isolation and in the presence of gene flow. Alternatively, H‐SS_gf_ would also apply if these lineages diverged with cycling periods of isolation in refugia followed by secondary contact and with repeated periods of introgression. Hypothesis H‐SS_i_ (Sonoran/Sinaloan isolation) assumes that these lineages diverged without introgression during a single event of isolation (physical or ecological) followed by secondary contact (see supporting information; Tables S1 and S2).

## Material and Methods

### Ethics statement

The University of Arizona Institutional Care and Use Committee (IACUC) approved all handling protocols (IACUC Control no. 09‐138).

### Samples

Phylogenetic analyses employed 82 DNA samples collected from 38 sites previously used in other studies (Edwards et al. 2004, 2010, 2015a, 2015b; Murphy et al. 2007). These represented samples from across the range of the desert tortoises (Table 1; Fig. 1). In addition, we obtained two samples of *G. berlandieri* from a private collection and two samples of *G. flavomarginatus* collected in Durango, Mexico (Morafka et al. 1994).

**Table 1:** Desert Tortoise sample locality information

Site ID	*n*	Taxa	Biotic community	Location	Site
UT	2	Goag‐ Northern	Mojave Desertscrub	Utah, USA	Near St. George
TC*	2	Goag‐ Northern	Mojave Desertscrub	Nevada, USA	Trout Canyon
SV	2	Goag‐ Northern	Mojave Desertscrub	California, USA	Shadow Valley
IV	2	Goag‐ Northern	Mojave Desertscrub	California, USA	Ivanpah
KH	1	Goag‐ Western	Mojave Desertscrub	California, USA	Kramer Hills
FIC	2	Goag‐ Western	Mojave Desertscrub	California, USA	Fort Irwin
ORC	1	Goag‐ Western	Mojave Desertscrub	California, USA	Ord‐Rodman
MCSH	1	Goag‐ Western	Mojave Desertscrub	California, USA	Marine Corps Air Ground Combat Center
MCB	1	Goag‐ Western	Mojave Desertscrub	California, USA	Marine Corps Air Ground Combat Center
CM	1	Goag‐ Western	Lower Colorado River Valley	California, USA	Chocolate Mountain Aerial Gunnery Range
FEN	1	Goag‐ Western	Mojave Desertscrub	California, USA	Fenner
G	1	Goag‐ Western	Mojave Desertscrub	California, USA	Goffs
CH	1	Goag‐ Western	Mojave Desertscrub	California, USA	Chemehuevi
UWV	2	Goag‐ Western	Mojave Desertscrub	California, USA	Upper Ward Valley
GS	2	Goag‐ Western	Ecotone: SDS/MDS	Arizona, USA	Southwest side of Black Mtns.
WBM	4	Admixed ‐ MOJ/SON	Ecotone: SDS/MDS	Arizona, USA	West side of Black Mtns.
EB	4	Admixed ‐ MOJ/SON	Ecotone: SDS/MDS	Arizona, USA	East Bajada Long Term Monitoring Plot
HSC	1	Admixed ‐ MOJ/SON	Ecotone: SDS/MDS	Arizona, USA	Hualapai Mtns.
HAR	2	Gomo ‐ SON	SDS: Arizona Upland	Arizona, USA	Harcuvar Mtns.
WM	4	Gomo ‐ SON	SDS: Arizona Upland	Arizona, USA	Wickenburg Mtns.
NW	1	Gomo ‐ SON	Ecotone: SDS/Lower Colorado River Valley	Arizona, USA	New Water Mtns.
EAG	2	Gomo ‐ SON	Ecotone: SDS/Lower Colorado River Valley	Arizona, USA	Eagletail Mtns.
SAU	2	Gomo ‐ SON	Ecotone: SDS/Lower Colorado River Valley	Arizona, USA	Sauceda Mtns. region
SL	3	Gomo ‐ SON	SDS: Arizona Upland	Arizona, USA	Sugarloaf, Mazatzal Mtns.
GRH	2	Gomo ‐ SON	SDS: Arizona Upland	Arizona, USA	Granite Hills
RK	2	Gomo ‐ SON	SDS: Arizona Upland	Arizona, USA	Saguaro National Park and adjacent land
SP	2	Gomo ‐ SON	SDS: Arizona Upland	Arizona, USA	San Pedro Valley
CAN	2	Gomo ‐ SON	Ecotone: SDS/Lower Colorado River Valley	Sonora, Mexico	La Candelaria
BAM	2	Gomo ‐ SON	Ecotone: SDS/Lower Colorado River Valley	Sonora, Mexico	Bamuri
TIB	4	Gomo ‐ SON	SDS: Central Gulf Coast	Sonora, Mexico	Tiburon Island
SER	2	Gomo ‐ SON	SDS: Central Gulf Coast	Sonora, Mexico	Seri region
RSJ*	2	Gomo ‐ SON	SDS: Plains of Sonora	Sonora, Mexico	San Judas
RM	3	Admixed ‐ SON/SIN	Ecotone: STS/SDS	Sonora, Mexico	Moscobampo
RAS	3	Admixed ‐ SON/SIN	Sinaloan Thornscrub	Sonora, Mexico	Arroyo Seco
OPO	4	Admixed ‐ SON/SIN	Sinaloan Thornscrub	Sonora, Mexico	Opodepe‐Las Milpas region
REA	3	Gomo ‐ SIN	Ecotone: TDF/STS	Sonora, Mexico	El Alamo
RLN	2	Gomo ‐ SIN	Ecotone: TDF/STS	Sonora, Mexico	La Noria
ALC	2	Gomo ‐ SIN	Tropical Deciduous Forest	Sonora, Mexico	Alamos‐Las Cabras
ALS*	2/2	Gomo ‐ SIN	Tropical Deciduous Forest	Sonora, Mexico	Alamos‐La Sierrita
FUE	4	Gomo ‐ SIN	Tropical Deciduous Forest	Sinaloa, Mexico	Rio Fuerte

Site ID corresponds with Figure 1; *n* = number of individuals sampled. Biotic community descriptions: TDF = Tropical Deciduous Forest, STS = Sinaloan Thornscrub, SDS = Sonoran Desertscrub and MDS = Mojave Desertscrub. Asterisk (*) indicates sites sampled for RNA‐seq (ALS samples used in both DNA and RNA analyses).

The RNA‐seq data gathering used nine individuals in three flowcell lanes on the Illumina HiSeq platform. We dedicated two lanes to high‐coverage sequencing of three individuals, one for each of the following lineages: a captive individual of *G. agassizii* in Arizona that originated in California (Moj_A haplotype); a captive individual of Sonoran *G. morafkai* from Arizona; and a wild‐caught Sinaloan *G. morafkai* obtained from just outside of Alamos, Sonora Mexico (Rancho Las Cabras; RLC). Raw data were used to assemble reference transcriptomes. The third flowcell lane was used to generate low‐coverage RNA‐seq reads from six samples that were then mapped to the reference assemblies to assess diversity within and among the three lineages. For these six samples, we hand‐captured and collected samples from wild desert tortoises from the following three sites in 2013: two Sinaloan *G. morafkai* from the Reserva La Sierrita, Sierra de Alamos, Sonora, Mexico (Fig. 1, site ALS; tropical deciduous forest); two Sonoran *G. morafkai* from the Rancho San Judas north of Hermosillo, Sonora, Mexico (Fig. 1, site RSJ; Sonoran desertscrub/plains of Sonora); and two *G. agassizii* from Trout Canyon west of Las Vegas bordering the Eastern Mojave and Northeastern Mojave recovery units (USFWS 2011), Clark County, Nevada (Fig. 1, site TC; Mojave desertscrub with *Larrea tridentata* and *Yucca brevifolia*).

For RNA sample collection, we obtained <1 mL whole blood via brachial or subcarapacial venipuncture and mixed it with a greater than 50% volume RNA lysis/binding buffer from the Ambion RNAqueous kit (Life Technologies, Thermo Fisher Scientific Inc., Grand Island, NY). Samples were immediately put on ice and then transferred to liquid nitrogen for storage within 4 h of collection. All RNA samples were verified as being of the assumed lineage (and not hybrids) with subsequent DNA analyses using 25 microsatellite loci and mtDNA (Edwards and Berry 2013).

### DNA sequencing

We sequenced a 1109 base pair (bp) portion of mitochondrial DNA (*ND3*, tRNA^arg^, *ND4L*, and part of *ND4*) following Edwards (2003) and Murphy et al. (2007). Some individuals sequenced for this locus had been used in previous studies (Murphy et al. 2007; Edwards et al. 2012, 2015a, 2015b), including the same fragment for *G. flavomarginatus* (Edwards et al. 2014). We optimized PCR conditions for six nuclear loci: *BDNF*,* R35*, and four uncharacterized loci identified by Thomson et al. (2008) derived from BAC libraries (TB02, TB07, TB53, and TB95). For PCR amplification of these loci, we used primer pairs as described by Leaché and McGuire (2006) and Thomson et al. (2008), except for *R35*, where we used a GenBank sequence (accession number AY434646) to design primers with OLIGO PRIMER ANALYSIS 6.68 (Molecular Biology Insights, Inc., Colorado Springs, CO) as follows: R35EX1_GOPH CACATACTGAATTTCCAGG, and R35EX2_GOPH GGACCTTTAAGTCATACAC.

We assessed optimal PCR conditions for each primer pair under 72 possible conditions by varying temperature from 52 to 64°C and MgCl_2_ concentration from 1.0 to 4.5 mmol/L. PCR amplifications for Sanger sequencing used 30 *μ*L reaction volumes containing 0.2 μmol/L of each primer, 10 mmol/L Tris–HCl (pH 8.3), 0.2 mmol/L of each dNTP, 0.4 units of Platinum Taq (Life Technologies, Thermo Fisher Scientific Inc.), 5.0 mmol/L KCl, 10 ng of genomic DNA, and locus‐specific amounts of MgCl_2_ (Table 2). PCR began with an initial 3 min denaturation at 94°C, followed by 35 cycles of 30 s at 94°C, 30 s at the locus‐specific annealing temperature (Table 2), and 90 s at 72°C, followed by 3 min incubation at 72°C. We submitted PCR product to the University of Arizona Genetics Core for DNA sequencing in both forward and reverse directions and followed standard protocols for the 3730XL DNA Analyzer (Applied Biosystems, Foster City, CA). We used CLC DNA WORKBENCH 5.7.1 (CLC bio, Denmark) to visually align sequences and DnaSP 5.10.01 (Librado and Rozas 2009) to build fasta files and generate general descriptive statistics. We used PHASE (Stephens and Donnelly 2003) for haplotype reconstruction of diploid loci.

**Table 2:** Summary of one mtDNA and four nDNA loci and their amplification conditions used for phylogenetic analysis of 86 tortoises in the genus *Gopherus*

Locus	# of haplotypes	Length (bp)	# variable sites	MgCl_2_ (mmol/L)	Annealing temp °C	Citation
mtDNA (ND3/ND4)	18	1109	190	4	52	Edwards 2003; Edwards et al. (2014)
BDNF	6	640	5	2	57	Leaché and McGuire (2006)
R35	13	500	17	4	53.4	Spinks et al. (2004)^a^
TB02	16	425	13	1	59	Thomson et al. (2008)
TB07	13	590	10	1.5	58	Thomson et al. (2008)

a Original primers from Fujita et al. (2004).

### Phylogenetic analysis

We reconstructed unrooted haplotype networks of nuclear loci to visualize relationships among lineages using BEAST 2.1.2 (Bouckaert et al. 2014). Substitution models were selected using MrModeltest 2.3 (Nylander 2004); all loci fit a HKY, gamma distribution with four discrete rate categories except TB07, which fit the GTR gamma distribution. BEAST analyses used a relaxed, log‐normal clock and the tree was calibrated using a Yule model (Drummond et al. 2006). We ran the Markov chain Monte Carlo (MCMC) for 500,000,000 generations sampling every 5000, with a burnin of 10%. We viewed results in TRACER 1.6.0 (Rambaut et al. 2003–2013) to ensure that the MCMC chains mixed well after the burnin and that ESS values were adequate (>100). We assessed patterns of haplotype diversity by grouping samples by species (*G. flavomarginatus, G. berlandieri, G. agassizii, G. morafkai*), by lineages within *G. morafkai* (Sonoran and Sinaloan) and by geographic regions where mtDNA differentiation had been previously observed (Murphy et al. 2007; Edwards et al. 2015a, 2015b) (Table S3).

For mitochondrial lineage reconstructions, we performed the analysis in BEAST as described above using *G. flavomarginatus* as the outgroup taxon to enable construction of a rooted tree. To establish estimates of time to most recent common ancestor (TMRCA) for the mtDNA locus only, we set the prior for our Bayesian analysis in BEAST for divergence time between *G. agassizii* and *G. morafkai* (Sonoran) lineages to 5.9 ± 0.5 Ma based on Edwards (2003). In addition, we used PAUP* 4.0b10 (Swofford 2002) to reconstruct maternal genealogies using both likelihood and parsimony optimality criterion searches to generate tree topologies. We compared these topologies with that derived from Bayesian analysis executed with BEAST. Analyses used unique haplotypes and all characters received equal weight. We performed a heuristic search with 100,000 random addition replicates. Support for inferred relationships was estimated using 10,000 nonparametric bootstrap replicates. We performed maximum likelihood analysis using the HKY model of nucleotide evolution.

We also performed maximum‐likelihood estimates using branch models of CODEML in PAML 4 (Yang 2007) to determine the mean selection pressures on different branches of the mtDNA tree. This method compared the ratio *d*_N_/*d*_S_, termed *ω*, where *ω *< 1 indicated purifying selection, *ω *= 1 indicated neutral selection, and *ω *> 1 indicated adaptive selection. First, we calculated *ω* under a one‐ratio model in which the same ratio occurred across the tree. Next, we estimated an independent *ω* value for each branch under the free‐ratio model.

We used the *BEAST model (Heled and Drummond 2010) for species tree estimation in BEAST using mtDNA and four of the nuclear loci (TB02, TB07, R35 and BDNF). *BEAST analyses used multilocus data and the multispecies coalescent approach to infer species trees. We assigned individuals to putative species/lineages, which was difficult for individuals of *G. morafkai* that occurred along the thornscrub/desertscrub ecotone zone (Edwards et al. 2015b). We defined individuals with questionable genotypes based on their mtDNA haplotype as either Sinaloan or Sonoran based on Edwards et al. (2015b). We used the HKY with four gamma distributed rate categories for all loci except TB07 which we applied the GTR with four gamma distributed rate categories. We used the Yule Process prior and did not set a coalescent prior. We set the population size function to linear with constant root (appropriate when the real population size dynamics tend to be continuous; Heled and Drummond 2008) and ran the MCMC for 500,000,000 generations with a 10% burnin for both strict and relaxed log normal clocks. We viewed results in Tracer; both runs achieved stationary MCMC distributions and effective sample size (ESS) values > 200. We used TreeAnnotator 1.7.5 (Rambaut and Drummond 2002–2012) to select the Maximum Clade Credibility tree that had the highest product of the posterior probabilities of all its nodes from the BEAST analysis, and FigTree 1.4.0 (Rambaut 2006–2012) to visualize the tree. We performed a qualitative analysis on consensus trees directly from the *BEAST trees file using DensiTree 2.2.1 (Bouckaert and Heled 2014).

### Next‐generation sequencing

We isolated total RNA from whole blood using standard protocols for the Qiagen RNeasy Kit (Qiagen, Valencia, CA). We quantified recovered RNA using a RiboGreen TBS‐380 Flourometer (Turner BioSystems, Sunnyvale, CA) and assessed sample quality with an Advanced Analytics Fragment Analyzer using the High Sensitivity RNA Kit (Advanced Analytics, Ames, IA). We used the Illumina TruSeq RNA kit (Illumina, Inc., San Diego, CA) to build the cDNA library from total RNA. The kit targeted polyadenylated mRNA for second strand cDNA synthesis and size‐selected via enzyme‐mediated fragmentation. While building the cDNA library, we applied unique tags to each individual sample. We quantified each cDNA library using a Kapa Biosystems qPCR Kit (Kapa Biosystems, Inc., Wilmington, MA) specific to the Illumina Adapter sequence. Next, we pooled samples in equimolar concentrations for the final cDNA library. We ran the three high coverage individuals representing each of the three tortoise lineages on two flowcell lanes using an Illumina HiSeq 2000 and we ran the six lower‐coverage individuals on a single flowcell lane using the Illumina HiSeq 2500 platform. All next‐generation sequencing protocols were performed at the University of Arizona Genetics Core following standard protocols.

### Transcriptome assembly

For each library, we processed raw reads to remove sequencing adaptors, trimmed for quality score (Q > 28) and length‐filtered (>37 bp) using TRIMMOMATIC 0.32 (Bolger et al. 2014). We used reads from the three high‐coverage samples (Mojave, Sonoran and Sinaloan) to create de novo transcriptome assemblies for each species/lineage, as well as a combined assembly consisting of reads from all three libraries to be used as a reference. We assembled transcript contigs using TRINITY (Grabherr et al. 2011; *Haas* et al. 2013) with default settings. As de novo transcriptome assemblies often consist of many thousands of possibly chimeric contigs that lack clear gene content (Cahais et al. 2012), we further filtered the TRINITY output for contigs with single gene annotations. To accomplish this, we treated the TRINITY contigs as a query in a BLASTX search of mouse and chicken proteins from UniProt (Magrane and UniProt Consortium 2011) with an E‐value cutoff of 1e‐6. We then selected contigs containing unique BLAST hits to incorporate into a reference transcriptome for downstream analyses.

We followed a slightly modified protocol of De Wit et al. (2012) for mapping and variant detection. We performed the analysis using the six low‐coverage RNA‐seq samples and mapped these to the reference transcriptome. We used Burroughs Wheeler Aligner 0.6.1 (Li and Durbin 2009) to generate Sequence/Alignment Map (SAM) files. We performed several trials to assess parameter sets and settled on using default parameters with assumed offset of 33, allowed for 0.005 differences between reference and query (‐n), and allowed up to five differences in the seed (‐k) to achieve > 67% of reads for each individual mapped to the reference transcriptome. We then converted SAM to BAM file format and removed duplicates using SamTools 0.1.18 (Li et al. 2009). Next, we merged all six individuals together to create a single BAM file and then followed recommendations in the De Wit et al. (2012) protocol to realign poorly mapped regions near indels using GenomeAnalysisTK‐1.0.5974 (GATK; McKenna et al. 2010). We also used GATK to detect and annotate variants and generate Variant Call Format (VCF) files (DePristo et al. 2011). We followed the recommendations of De Wit et al. (2012) and called only variant sites with a Phred scale quality of more than 30. We then performed low threshold variant detection and Variant Quality Score Recalibration (VQSR) following De Wit et al. (2012) to build a Gaussian mixture model to be able to accurately distinguish true variant sites from false positives. Subsequently, we parsed out only the variant sites for which we have genotype information for all individuals with a Phred quality score cutoff of 20. We used these data for all downstream analysis.

We performed Principal Components Analysis as an initial assessment of these data using SMARTPCA in EIGENSOFT 5.0.2 (Patterson et al. 2006). We then annotated these data using TransDecoder (Haas et al. 2013) and identified candidate coding regions within transcript sequences. TransDecoder generated a gff3 file which we then converted to a GTF (general transfer format) file using GFFREAD in CUFFLINKS 2.2.1 (Trapnell et al. 2010). Next, we used SNPdat 1.0.5 (Doran and Creevey 2013) to generate a GTF file annotating gene sequences shared by all six individuals.

### Demographic inference using ∂a∂i

We used ∂a∂i 1.6.3 (Gutenkunst et al. 2009) to fit demographic models to our six transcriptome samples (see supporting information; Tables S1 and S2). In addition, the ∂a∂i analysis generated a species tree for the three taxa independent of the multi‐locus Bayesian analysis (Table S2). We built the folded joint allele frequency spectrum using only variants that SNPdat unambiguously called as synonymous and that were successfully genotyped in all six individuals. To choose between our final 5‐ and 6‐parameter models, we performed a likelihood ratio test (LRT) with a statistical correction for linkage based on the Godambe information matrix (Coffman, Hsieh, Gravel, & Gutenkunst, *pers comm*). Parameter confidence intervals were estimated by 100 conventional bootstraps over contigs and calculated as $\overset{¯}{\theta }\pm 1.96\times \sigma (\theta )$, where *θ* was the parameter of interest and *σ* was the standard deviation of the bootstrap results. To convert parameters from population genetics units (scaled by ancestral population size *N*_*a*_) to physical units, we assumed a divergence time between *G. agassizii* and Sonoran *G. morafkai* lineages of 5.9 Ma (Edwards 2003) and a generation time of 25 years (USFWS 1994). To convert bootstrap parameter values, we used our maximum composite‐likelihood parameter values and the relation *θ *= 4*N*_*a*_ *μ*L to estimate the quantity *μ*L and then applied this value all bootstrap samples.

## Results

We obtained usable sequences for the mtDNA and four of the six nuclear loci (*BDNF*,* R35*, TB02 and TB07; Table 2). Loci TB53 and TB95 generated electropherograms with excessive noise on repeated attempts, and thus they were excluded. In phasing the nuclear DNA data, all samples for *BDNF* had a minimum pair probability of 0.996 and all were selected for downstream analyses. For R35, all but two samples were selected with a minimum pair probability of 0.942. For TB02, all samples were accepted; three pairs fell below a probability of 0.922 but were still included. None of these samples had unique haplotypes that were not represented in another sample. For TB07, 51 samples had 100% pair probabilities and were selected for analysis; 18 samples had pair probabilities close to 0.50 between two pairs of haplotypes (13 samples with the same two possible pairs of haplotypes involved *G. agassizii* or Sonoran‐type *G. morafkai*; five samples of *G. morafkai* collected in Mexico shared the same two ambiguous pairs). Both examples represented the same ambiguity in the same SNP and each of the four haplotypes among the ambiguous pairs was represented in the other 51 samples. For this locus we choose the pair with the highest *P* value to use for downstream analyses.

**Figure 2:**
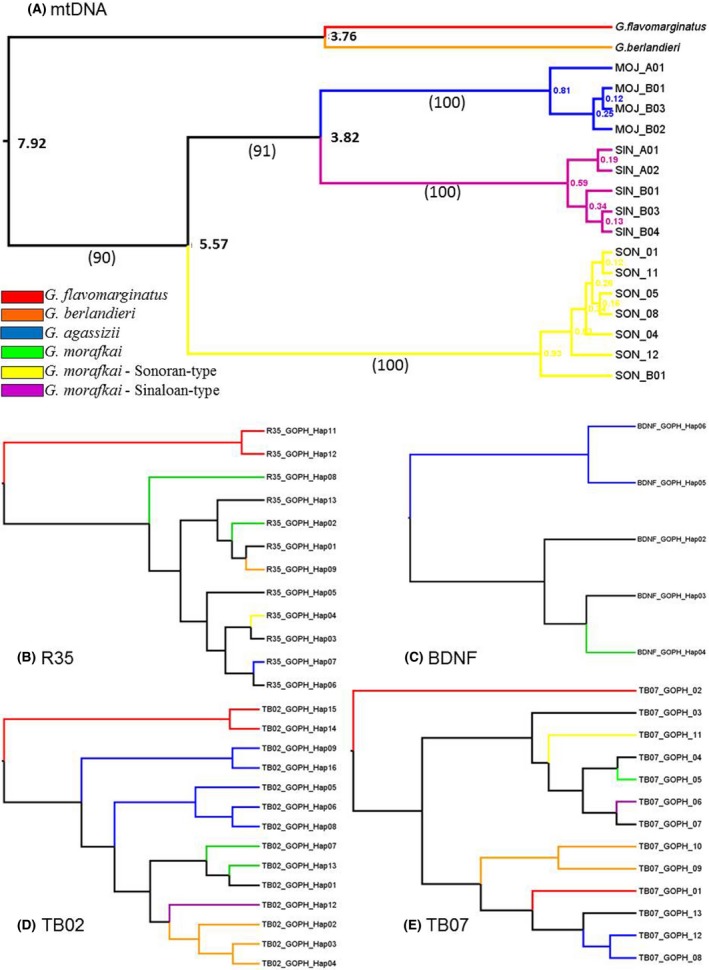
Bayesian reconstructions of representative nuclear and mtDNA haplotypes using BEAST; Colored branches indicate haplotypes fixed in designated lineages. (A) mtDNA nodes labeled with estimated time to most recent common ancestor (TMRCA) in millions of years. Bootstrap values in parentheses estimated from parsimony reconstruction. (B–E) nDNA allele networks rooted at midpoint of the greatest distance to present tree‐like associations.

Among the nuclear loci, haplotypes were both lineage‐specific and found globally across species of *Gopherus* (Table S3, Fig. 2). Haplotype diversity, nucleotide polymorphism, and nucleotide diversity estimates varied across loci and among‐sample groupings and did not suggest strong trends. For example, haplotype diversity was greater in *G. agassizii* then *G. morafkai* at some loci but not others, and Tajima's D was positive for some loci and negative for others (Table S4).

### Phylogenetic analysis

**Figure 3:**
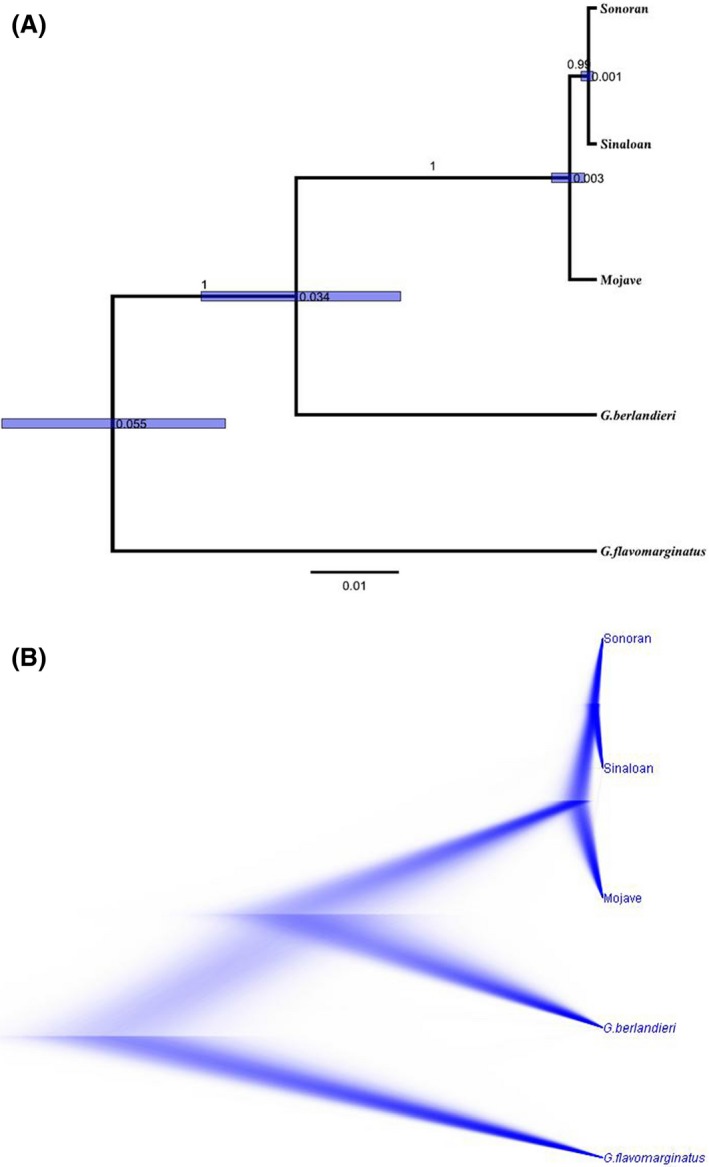
Estimated species tree from multilocus data using *BEAST reconstructed from mtDNA and 4 nDNA loci from 86 tortoises in the genus *Gopherus*;* G. agassizii*, Mojave lineage; *G. morafkai* Sonoran and Sinaloan lineages; *G. berlandieri*;* G. flavomarginatus*. Generated from 500 million iterations using a strict clock; (A) Maximum clade credibility tree with common ancestor node heights with nodes representing relative TMRCA (scale bar in arbitrary units), bars corresponding with standard deviations and branches labeled with posterior probabilities, and (B) DensiTree visualization of consensus trees.

The matrilineal genealogy (mtDNA tree) had strong support across analyses for distinct Mojave/Sonoran/Sinaloan matrilines. Notwithstanding, the geographically distant Mojavian and Sinaloan matrilines were resolved as sister taxa, and together they were the sister group to the intervening Sonoran matriline (Fig. 2A). The multilocus analyses exhibited the same topology for both strict and relaxed log normal clocks with species relationships consistent but with the important expectation that the adjacent Sinaloan and Sonoran matrilines clustered together and formed the sister group of the Mojave. Estimated TMRCAs exhibited wide standard deviations (Fig. 3). Qualitative analysis using DensiTree suggested strong concordance among iterations for the resulting tree topology but with less precision around depth of nodes (Fig. 3B).

For tests of selection on the mtDNA locus, our estimations of *ω* employed different models for branches of the tree (Table 3). First, assuming a uniform *ω* for all branches of the five species/lineages of *Gopherus*,* ω* was estimated to be 0.6192, which was significantly less than one (*P *<* *0.05). This result suggested that this gene was under overall strong purifying selection. Next, we applied a model in which every branch had its own *ω*. This model was significantly more effective than one‐ratio model (*P *<* *0.05; Table 3), suggesting that *ω* varied among the different lineages. The value of *ω* on the branch leading to SIN (Sinaloan lineage haplogroup) was estimated to be larger than one (1.1), indicating that positive selection may have affected this lineage (Table 3).

**Table 3:** Likelihood ratio tests for selection pressure estimated among mtDNA haplotypes from tortoises in the genus *Gopherus*

Selection models	np^a^	lnL^b^	*ω* (d_N_/d_S_)	Models compared	*P* values
A. All branches have one *ω*	9	−2463.7	*ω *= 0.62		
B. All branches have the same *ω* = 1	8	−2469.4	*ω* = 1	A vs. B	<0.001
C. Each branch has its own *ω*	15	−2454.6	Variable *ω* by branch	A vs. C	<0.01

a Number of parameters.

b The natural logarithm of the likelihood value.

### Network analysis

Our network reconstructions of alleles (Fig. 2) used midpoint rooting of the greatest distance, and not outgroup analysis, to present tree‐like associations. As such, the terminals are alleles, and not individuals. Allelic associations of individuals were listed in Table S3. In one locus, *BDNF*, all species of tortoises shared most alleles. In contrast, unique alleles were constrained to species of desert tortoises in *R35*, TB02 and TB07, although a few alternative and presumably primitive alleles occurred in more than one species.

### Transcriptome assembly

**Figure 4:**
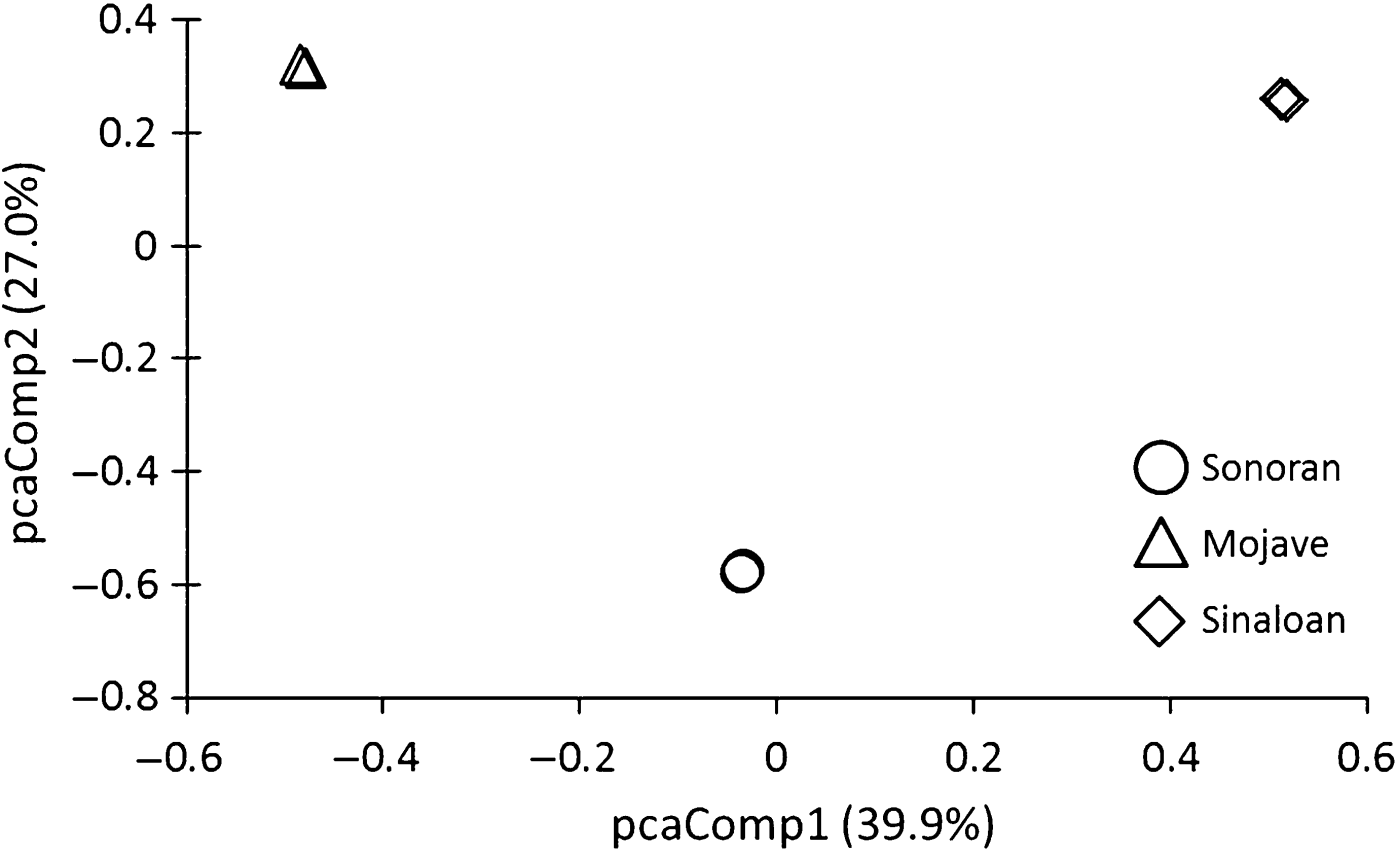
Principal Components Analysis of 95,220 polymorphic sites in the reference transcriptome among six individuals representing the three lineages of desert tortoise; Mojave, Sonoran, and Sinaloan.

We assembled 111,635,751 trimmed reads from whole blood total RNA into a combined *G. agassizii* and *G. morafkai* assembly that contained 235,412 contigs (Table 4). The blast‐filtered combined assembly contained 40,341 transcripts with a contig N50 of 3010 bp and a mean contig length of 1957 bp. After aligning the six individuals against the combined assembly and identifying the variant alleles, we characterized 95,220 polymorphic sites for which we have genotype information for all individuals. The PCA assessment showed extremely strong clustering of individuals within each lineage and relatively equidistant differentiation among lineages (Fig. 4).

**Table 4:** Summary of RNA‐seq and reference transcriptome assembly results for three individuals representing each lineage of desert tortoise

Trinity assembly	No. reads	No. transcripts	N50	Mean contig length (bp)
*G. agassizii* (Mojave)	44,068,129	150,135	1190	709
*G. morafkai* (Sonoran)	22,528,007	202,778	2500	1093
*G. morafkai* (Sinaloan)	45,039,615	138,380	1994	899
Combined assembly	111,635,751	235,412	1302	718
Combined filtered assembly	–	40,341	3010	1957

### Demographic modeling

We used the allele frequency spectrum (AFS)‐based inference tool ∂a∂i (Gutenkunst et al. 2009) to infer the joint demographic history of the three lineages of desert tortoise from our transcriptomes. Because AFS‐based demographic inference was shown to be sensitive to genotyping errors (Gutenkunst et al. 2009) and selection (Williamson et al. 2005), we considered only synonymous variants successfully called in all six individuals, yielding an AFS with 20,126 synonymous variants from 7665 contigs.

To guide development of three‐population models, we first considered simpler two‐population models. Initial two‐population models without gene flow (modeling allopatric speciation) consistently yielded a larger effective population size for the Sonoran population than the others and a more recent divergence between the Sinaloan and Sonoran populations than between either of those populations and the Mojave (Table S1). Consistent with this result, the best‐fitting three population models involved recently diverged Sinaloan and Sonoran populations (Table S2). This result provided independent support for the *BEAST species tree. When we added gene flow (H‐MS_gf_, H‐SS_gf_) into the models, either as continuous flow, such as through parapatric speciation with continuous contact, or delayed flow, such as resulting from cycling periods in refugia followed by introgression during secondary contact, the maximum composite‐likelihood estimates for the gene flow parameter converged to zero (Table S1). Thus, we found no evidence of gene flow between any pair of the three populations and, thus, rejected hypotheses H‐MS_gf and_ H‐SS_gf_.

**Figure 5:**
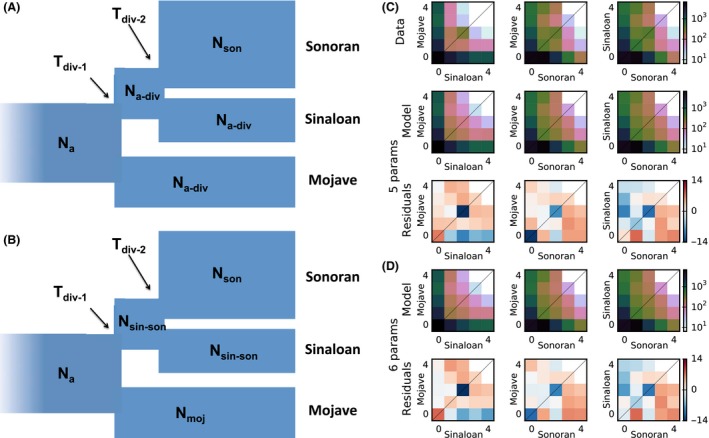
Best‐fit demographic models and observed and predicted frequency spectra for Mojave, Sinaloan, and Sonoran desert tortoise populations. (A) Simpler model fit with the five free parameters labeled. (B) More complex model, with the 6 free parameters labeled. (C, D) The marginal spectra for each pair of populations. Row one is data, rows two and four are models, and rows three and five are Anscombe residuals of model minus data. Ns represent effective population sizes and Ts represent times of population divergence.

Based on our two‐population analyses, we considered three‐population models in which the Sinaloan and Sonoran populations were sisters and there was no gene flow between them. Figure 5 showed the two best‐fit models among those we tested (composite log‐likelihood: −887 for the 6‐parameter model vs. −909 for the 5‐parameter model). While the two models had the same tree topology, the 6‐parameter model had an additional free parameter for the effective population size of the contemporary Mojave population. Qualitatively, these models produced similar allele frequency spectra and residuals when compared with the data (Fig. 5C,D), but the 6‐parameter was preferred in a composite likelihood ratio test (adjusted likelihood ratio 9.242, *P *=* *0.0024, chi‐squared test with df = 1). To estimate parameter uncertainty while accounting for linkage among variants, we used conventional bootstrapping. Table 5 showed the confidence intervals for the parameters of our 6‐parameter demographic model. This best‐fit model suggested that the Mojave and Sinaloan populations have similar effective sizes (128,000 and 150,000 individuals, respectively), but the effective size of the Sonoran population is much larger (600,084 individuals). The two divergence times in our model are also similar (Table 5), suggesting a trichotomy among these populations.

**Table 5:** Maximum composite likelihood parameter estimates and confidence intervals for the best‐fit 6‐parameter demographic model in Figure 5B. Parameters are effective population sizes (*N*) in individuals and times of divergence (T) in years

Demographic parameter	Estimate	95% C.I.
N_a_: size of *Gopherus* ancestral population	336,200	328,000–344,000
N_moj_: size of contemporary Mojave population	128,400	122,000–135,000
N_sin‐son_: size of contemporary Sinaloan population	149,600	143,000–156,000
N_son_: size of contemporary Sonoran population	600,000	548,000–668,000
T_div‐1_: time of Mojave divergence	5,900,000	5,597,000–6,183,000
T_div‐2_: time of Sinaloan and Sonoran divergence	5,650,000	5,376,000–5,967,000

## Discussion

### Phylogenetic relationships

The multilocus Bayesian species tree depicts genetically distinct Sonoran, Sinaloan, and Mojave lineages of desert tortoise. This tree depicts Sonoran and Sinaloan tortoises as sister lineages (Fig. 3) nested as the sister group of the Mojave lineage, which is expected given their geographic distributions (Fig. 1). While the topology of the species tree is robust, the branch lengths are not likely representative of true divergence times. The branch lengths may be distorted by differences in mean selection pressure among branches, as the PAML analysis indicated for the mtDNA locus. Because we cannot estimate the actual mutation rate of each locus, we must rely on the well‐established date of the Bouse inundation that caused the vicariant divergence of *G. agassizii* and *G. morafkai*. This inundation now forms the Colorado River boundary between the species (Avise et al. 1992). Notwithstanding, mtDNA mutation rates based on this geological event are inconsistent with fossil records of divergence among other congeners. The molecular estimates of divergence within *Gopherus* may be too recent (Avise et al. 1992; Bramble and Hutchison 2014). Until a recalibration of the existing molecular clock is performed using distantly related groups, we consider our projected evolutionary rates for desert tortoises (Figs 2 and 3) to be conservative.

### Patterns of divergence

The RNA‐seq analyses yield two results that test our hypotheses. First, the best‐fit model in the ∂a∂i analysis for the relationship among the three taxa (Fig. 5 and Table S2) is concordant with the *BEAST species tree (Fig. 3) but more clearly elucidates the relative divergence times among the three lineages. The ∂a∂i result suggests that the Sonoran/Sinaloan split occurred only a short time after (or simultaneous with) the divergence of *G. agassizii* (Table 5). Thus, the three lineages form a trichotomy with relatively equal times of divergence from each other (Fig. 5).

The ∂a∂i analysis also finds no evidence of gene flow during divergence of the Sonoran/Sinaloan lineages. Despite strong biogeographic evidence that the current contact zone between the Sonoran and Sinaloan lineages is a result of secondary contact after periods of isolation in Pleistocene refugia (Edwards et al. 2015b) the rejection of hypothesis H‐SS_gf_ suggests that divergence in parapatry and/or periods of secondary contact did not result in significant introgression between lineages. The best fit model for Sonoran/Sinaloan divergence (e.g. no migration) does not differ from that obtained for the allopatric divergence of Sonoran/Mojave. Thus we cannot reject hypothesis H‐SS_i_ that Sonoran and Sinaloan lineages diverged without significant introgression and that isolation (geographical or ecological) is responsible for the divergence. Secondary contact followed the isolation, as is currently observed.

Lineages of desert tortoises have similar (and simultaneous) processes of speciation. Either geography (geographic distance and physical barriers) or selection through ecological niche segregation should drive divergence via a reduction in gene flow, and both drivers may occur simultaneously (Endler 1977; Cooke et al. 2014). *Gopherus agassizii* appears to have diverged first as a result of allopatry. The secondary contact zone in northwestern Arizona shows that *G. agassizii* adapted over time to the unique environmental conditions of the Mojave Desert (Edwards et al. 2015a). Differential adaptation may occur in allopatry and, thus, ecological speciation does not necessarily require sympatry (Bernardi 2013).

Geographic isolation may also explain speciation of the Sonoran and Sinaloan lineages and an allopatric ‘speciation pump’ (April et al. 2013) may have facilitated divergence. No obvious geographical barrier explains their long‐term isolation. One alternative explanation, and one that better fits the geographical history of the region, is that the Sonoran and Sinaloan lineages first diverged into distinct ecotypes under a parapatric model of speciation during the Neogene Period. This scenario requires isolation in ephemeral Pleistocene refugia after the lineages differentiated (Fisher‐Reid et al. 2013). Our results fail to find a genetic signature of ecological isolation (parapatric model) and in doing so this scenario cannot be differentiated from the allopatric model.

A growing number of empirical examples suggest that speciation can occur without spatial separation, particularly in the case of ecologically driven selection (Rundle and Nosil 2005; Pinho and Hey 2010; Smadja and Butlin 2011). Our assumption that signatures of ancient admixture between Sonoran and Sinaloan lineages would be identifiable relies on two conditions: (1) the likelihood of recurring biogeographic proximity during their evolution and (2) observations of contemporary hybridization. However, it may be that there are unique circumstances under which a signature of past gene flow will remain in the genome and these conditions were not met during the parapatric divergence of Sonoran and Sinaloan lineages of *G. morafkai*. Selection tends to favor divergence in the presence of gene flow only when a few traits or genes are involved, or when extensive pleiotropy exists (Smadja and Butlin 2011). Detection of past signatures of neutral introgression requires sufficient time for advantageous alleles to attain high frequency or fixation (e.g., time to fixation). In addition, the strength and timing of gene flow influences the likelihood of speciation (Kisel and Barraclough 2010; Pinho and Hey 2010; Smadja and Butlin 2011). Thus, even when divergence between sympatric taxa occurs, signals of past introgression and any remaining genetic signature may not remain or might constitute only a very minor portion of the existing genome (Mendez et al. 2012).

Our RNA‐seq analyses involve six samples only and these are limited to discrete populations. However, the analyses include a massive amount of independent gene sequences and these allow for the high resolution of evolutionary patterns. Our sampling strategy minimizes geographic bias via equidistant sampling, while maximizing the opportunity to detect introgression in the genome. Short‐read technology ensures that the number of loci does not limit our analysis (Table 4) and we present a high level of resolution beyond what could be inferred through traditional analyses. Importantly, analyses of the RNA‐seq data effectively test our hypotheses, and we are confident that these results reflect the evolutionary history of the desert tortoise.

Inferences based on large numbers of gene sequences and few individuals have been shown to be robust for inference of population history (Wang and Hey 2010; Lohse et al. 2011; Jones et al. 2012; Hearn et al. 2014). Robinson et al. (2014) used simulations to test the ability of ∂a∂i to differentiate between models of population divergence with and without gene flow. In particular, their simulations included cases very similar to our study, with two individuals sampled per population, 13,000–18,000 SNPs analyzed, and a divergence time of *T* = 0.25. They found that ∂a∂i confidently distinguished between models with zero or moderate migration between two populations (median Akaike weight for the true model at least 0.9). These results suggest that if substantial gene flow had occurred between lineages during the evolution of desert tortoises, we would detect it.

For species of conservation concern, like desert tortoises, it is necessary to sample only a small amount of tissue using non‐lethal methods. Thus, whole blood provides the most obtainable source of DNA for our study (Meitern et al. 2014). It is difficult to assess whether the number of putative transcripts in our analysis or the number of polymorphic sites characterized meets expectations because this study focuses on a nonmodel organism. Our assembly obtains almost twice as many functionally annotated transcripts than obtained from whole blood RNA‐seq of greenfinch (*Carduelis chloris*), another nonmodel organism (Meitern et al. 2014). The assembly of de novo genomes has multiple challenges (McGettigan 2013) and we expect a high likelihood of generating technical artifacts. We address this concern through careful screening (UniProt queries) and the implementation of very conservative filtering. Regardless, these data may contain a low proportion of sequencing and assembly errors. Thus, our data appear more than adequate for the purpose of phylogenetic reconstruction.

Our small sample size precludes meaningful selection tests, which could identify the genomic regions driving the observed evolutionary patterns. Notwithstanding, our analyses clarify the evolutionary history of these tortoises and yield insights into the relative contributions of isolation and gene flow to the formation of these species. It also opens up many new opportunities for studying speciation in a natural setting. Further research, with the aid of an annotated genome, may investigate the presence of chromosomal rearrangements, which may better explain the mechanism by which distinct species persist despite the potential for hybridization (Kulathinal et al. 2009). Future research might also examine heterogeneity among loci in proportion to levels of divergence, which could shed additional light on the debate over the formation of ‘genomic islands’ in the process of speciation (Feder and Nosil 2010; Nachman and Payseur 2012; Cruickshank and Hahn 2014).

## Conflict of Interest

None declared.

## Data Archival Location

All mtDNA and nDNA *gene sequence data are available* at GenBank; Accession nos.: DQ649408.1, DQ649409.1, DQ649394.1, DQ649398.1–DQ649401.1, DQ649404.1, DQ649405.1, and KM411506–KM411571.

RNA‐seq sequence data archived in the NCBI Sequence Read Archive (SRA); BioProject ID 281763.

Transcriptome assembly.fasta file, SNP data.vcf, and shared genotype.txt file used for this publication have been archived in Dryad. Provisional DOI: doi:10.5061/dryad.g0m5s.

## Supporting information


**Table S1.** Models evaluated during demographic inference using ∂a∂i 1.6.3 (Gutenkunst et al. 2009).
**Table S2.** Inference of species tree topology among the three tortoise populations.
**Table S3.** Haplotype summary of mtDNA and 4 nDNA loci.
**Table S4**. Overview of DNA polymorphism for mtDNA and 4 nDNA loci estimated for tortoises in the genus *Gopherus*.
