# Supplementary material for: Assessing models of speciation under different biogeographic scenarios; an empirical study using multi‐locus and RNA‐seq analyses
Source: Ecol Evol. 2016 Jan 7;6(2):379–96. doi: 10.1002/ece3.1865 (PMC4729248; doi:10.1002/ece3.1865)
Supplement: Supplementary file 1 — Table S1. Models evaluated during demographic inference using ∂a∂i 1.6.3 (Gutenkunst et al. 2009). Table S2. Inference of species tree topology among the three tortoise populations. Table S3. Haplotype summary of mtDNA and 4 nDNA loci. Table S4. Overview of DNA polymorphism for mtDNA and 4 nDNA loci estimated for tortoises in the genus Gopherus. [file ECE3-6-379-s001.doc]

**Supporting Information**

**Assessing models of speciation under different biogeographic scenarios; an empirical study using multi-locus and RNA-seq analyses**

Taylor Edwards, Marc Tollis, PingHsun Hsieh, Ryan N. Gutenkunst, Zhen Liu, Kenro Kusumi, Melanie Culver, Robert W. Murphy

**Table S1. Models evaluated during demographic inference using ∂a∂i 1.6.3 (Gutenkunst *et al.* 2009). Parameter N is effective population size, T_div_ is Time of Split (in units of 2N_a_ generations, where N_a_ is the ancestral effective population size), M is migration rate (in units of 2N_a_), T_M_ is Time of Migration after Isolation (in units of 2N_a_ generations), and f is admixture proportion.**

| **Model** | **Parameters** | **Population pair X-Y: Log-likelihood** | **[ Parameter Values]** | **Optimization** |
| --- | --- | --- | --- | --- |
| 2D – Simple Split | 4 parameters  [N_a_, Nx, Ny, T_div_] | Son-Sin: -153.6  Son-Moj: -142.3  Sin-Moj : -189.3 | [1, 1.160, 0.409, 0.279]  [1, 1.649, 0.403, 0.339]  [1, 0.435, 0.343, 0.343] | All converged. |
| 2D – Simple Split with symmetrical migration | 5 parameters  [Na, Nx, Ny, T_div_, M] | Son-Sin: -153.6  Son-Moj: -142.3  Sin-Moj: -189.3 | [1, 1.158, 0.409, 0.279, *0.0001]  [1, 1.648, 0.403, 0.339, *0.0001]  [1, 0.435, 0.343, 0.342, *0.0001] | *: The migration (M) parameters all converged to the defined lower bound of 1x10^-4^.  Note: For the asymmetrical migration model with parameters M_X->Y_ and M_Y->X_, in all cases both M_X->Y_ and M_Y->X_ converged to the defined lower bound of  1x10^-4^. |
| **Model** | **Parameters** | **Population pair X-Y: Log-likelihood** | **[ Parameter Values]** | **Optimization** |
| 2D – Simple Split  isolation, followed by symmetrical migration | 6 parameters  [N_a_, N_X_, N_Y_, T_div_, T_M_, M] | Son-Sin: -153.6  Son-Moj: -142.3  Sin-Moj: -189.3 | [1, 1.158, 0.409, 0.140, **0.398, *0.0001]  [1, 1.648, 0.403, 0.169, **0.124, *0.0001]  [1, 0.435, 0.342, 0.171, **0.134, *0.0001] | *: All migration parameters converged to the defined lower-bound of 1x10^-4^.  **: Times of migration onset did not converge.  Note: For asymmetrical migration model, with parameters M_X->Y_ and M_Y->X_, both M_X->Y_ and M_Y->X_ always converged to the defined lower bound and the parameter of initial time of gene flow also fail to converge. |
| 2D – Simple Split with symmetrical admixture | 5 parameters  [N_a_, N_X_, N_Y_, T_div_, T_f_, f] | Son-Sin: -153.6  Son-Moj: -142.3  Sin-Moj: -189.3 | [1, 0.093, 0.038, 0.085, 0.023, 0.909]  [1, 0.902, 0.281, 0.513, 0.209, 0.706]  [1, 0.318, 0.283, 0.616, 0.261, 0.717] | Parameters failed to converge.  Note: In the asymmetrical admixture model with parameters f_X->Y_ and f_Y->X_, both f_X->Y_ and f_Y->X_ failed to converge. |

**Table S2: Inference of species tree topology among the three tortoise populations.** Both models contain only four parameters, and the last divergence event creates two populations (faded, popX and popY) that have the same configuration of allele frequencies as their common ancestor (popX-popY). (A) Species tree in which the outgroup is the Mojave population. (B) Species tree in which the outgroup is the Sonoran population. (B) Species tree in which the outgroup is the Sinaloan population. Model parameters follow the similar definitions described in Table S1.

| **Model** | **Parameters** | **Log-likelihood** | **[ Parameter Values]** | **Optimization** |
| --- | --- | --- | --- | --- |
| 3D- Simple split | 4 Parameters  [Na, N_moj_, N_sin-son_, T_div_] | -7472 | [1, 0.384, 1.596, 0.356] | Converged |
| 3D- Simple split | 4 Parameters  [Na, N_sin_, N_son-moj_, T_div_] | -8087 | [1, 0.471, 1.404, 0.356] | Converged |
| 3D- Simple split | 4 parameters  [Na, N_son_, N_sin-moj_, T_div_] | -14415 | [1, 1.226, 0.99, 0.255] | Converged |

**Table S3. Haplotype summary of mtDNA and 4 nDNA loci**. Taxa grouped by species and geographic distribution, where *n* = number of individuals sequenced. Parenthetical values represent number of haplotypes assigned for individuals with ambiguous haplotype determination.

| A. mtDNA |  | Haplotypes (with GenBank accession numbers) | | | | | | | | | | | | | | | | | |
| --- | --- | --- | --- | --- | --- | --- | --- | --- | --- | --- | --- | --- | --- | --- | --- | --- | --- | --- | --- |
| Taxa | *n* | *G. berlandieri*  DQ649409.1 | *G. flavomarginatus*  DQ649408.1 | MOJ_A01  DQ649394.1 | MOJ_B01  DQ649398.1 | MOJ_B02  DQ649399.1 | MOJ_B03  DQ649400.1 | SON_01  DQ649401.1 | SON_04  DQ649404.1 | SON_05  DQ649405.1 | SON_08  KM411515 | SON_11  KM411518 | SON_12  KM411519 | SON_B01  KM411521 | SIN_A01  KM411506 | SIN_A02  KM411507 | SIN_B01  KM411510 | SIN_B03  KM411512 | SIN_B04  KM411513 |
| *G. flavomarginatus* | 2 |  | 2 |  |  |  |  |  |  |  |  |  |  |  |  |  |  |  |  |
| *G. berlandieri* | 2 | 2 |  |  |  |  |  |  |  |  |  |  |  |  |  |  |  |  |  |
| *G. agassizii* - Western | 12 |  |  | 12 |  |  |  |  |  |  |  |  |  |  |  |  |  |  |  |
| *G. agassizii* - Northern | 6 |  |  |  | 3 | 1 | 2 |  |  |  |  |  |  |  |  |  |  |  |  |
| *G. agassizii* - Arizona | 10 |  |  | 10 |  |  |  |  |  |  |  |  |  |  |  |  |  |  |  |
| *G. morafkai* - Sonoran, Arizona | 21 |  |  |  |  |  |  | 16 | 1 | 4 |  |  |  |  |  |  |  |  |  |
| *G. morafkai* - Sonoran, Mexico | 10 |  |  |  |  |  |  | 6 |  |  | 1 | 2 | 1 |  |  |  |  |  |  |
| *G. morafkai* - Sonoran, ecotone | 6 |  |  |  |  |  |  | 5 |  |  |  |  |  | 1 |  |  |  |  |  |
| *G. morafkai* - Sinaloan, ecotone | 7 |  |  |  |  |  |  |  |  |  |  |  |  |  | 6 | 1 |  |  |  |
| *G. morafkai* - Sinaloan | 10 |  |  |  |  |  |  |  |  |  |  |  |  |  | 4 | 2 | 2 | 1 | 1 |
| Total | 86 | 2 | 2 | 22 | 3 | 1 | 2 | 27 | 1 | 4 | 1 | 2 | 1 | 1 | 10 | 3 | 2 | 1 | 1 |

| B. R35 |  | Haplotype | | | | | | | | | | | |
| --- | --- | --- | --- | --- | --- | --- | --- | --- | --- | --- | --- | --- | --- |
| Taxa | *n* | Hap 01  KM411530 | Hap 02  KM411531 | Hap 03  KM411532 | Hap 04  KM411533 | Hap 05  KM411534 | Hap0 6  KM411535 | Hap 07  KM411536 | Hap 08 KM411537 | Hap 09  KM411538 | Hap 11  KM411540 | Hap 12  KM411541 | Hap 13  KM411542 |
| *G. flavomarginatus* | 1 |  |  |  |  |  |  |  |  |  | 1 | 1 |  |
| *G. berlandieri* | 1 |  |  |  |  |  |  |  |  | 2 |  |  |  |
| *G. agassizii* - Western | 10 |  |  | 1 |  | 18 | 1 |  |  |  |  |  |  |
| *G. agassizii* - Northern | 6 |  |  | 2 |  | 2 | 4 |  |  |  |  |  | 4 |
| *G. agassizii* - Arizona | 10 | 3 |  |  |  | 15 | 1 | 1 |  |  |  |  |  |
| *G. morafkai* - Sonoran, Arizona | 21 | 30 |  | 7 | 3 | 2 |  |  |  |  |  |  |  |
| *G. morafkai* - Sonoran, Mexico | 9 | 9 | 6 | 2 |  | 1 |  |  |  |  |  |  |  |
| *G. morafkai* - Sonoran, ecotone | 6 | 4 |  |  |  | 2 |  |  | 3 |  |  |  | 3 |
| *G. morafkai* - Sinaloan, ecotone | 6 | 5 | 1 | 1 |  |  |  |  | 4 |  |  |  | 1 |
| *G. morafkai* - Sinaloan | 9 | 3 |  |  |  | 1 | 2 |  | 9 |  |  |  | 3 |
| Total | 79 | 54 | 7 | 13 | 3 | 41 | 8 | 1 | 16 | 2 | 1 | 1 | 11 |

| C. BDNF |  | Haplotype | | | | |
| --- | --- | --- | --- | --- | --- | --- |
| Taxa | *n* | Hap02  KM411525 | Hap 03  KM411526 | Hap 04 KM411527 | Hap 05  KM411528 | Hap0 6  KM411529 |
| *G. flavomarginatus* | 2 |  | 4 |  |  |  |
| *G. berlandieri* | 2 | 3 | 1 |  |  |  |
| *G. agassizii* - Western | 9 |  | 8 |  | 6 | 4 |
| *G. agassizii* - Northern | 4 |  | 1 |  | 7 |  |
| *G. agassizii* - Arizona | 10 |  | 7 |  | 9 | 4 |
| *G. morafkai* - Sonoran, Arizona | 21 | 10 | 32 |  |  |  |
| *G. morafkai* - Sonoran, Mexico | 9 | 6 | 12 |  |  |  |
| *G. morafkai* - Sonoran, ecotone | 6 |  | 10 | 2 |  |  |
| *G. morafkai* - Sinaloan, ecotone | 6 | 1 | 9 | 2 |  |  |
| *G. morafkai* - Sinaloan | 10 | 3 | 14 | 3 |  |  |
| Total | 79 | 23 | 98 | 7 | 22 | 8 |

| D. TB02 |  | Haplotype | | | | | | | | | | | | | |
| --- | --- | --- | --- | --- | --- | --- | --- | --- | --- | --- | --- | --- | --- | --- | --- |
| Taxa | *n* | Hap 01  KM411556 | Hap0 2  KM411557 | Hap0 3  KM411558 | Hap0 4  KM411559 | Hap 05  KM411560 | Hap 06  KM411561 | Hap0 7  KM411562 | Hap0 8  KM411563 | Hap 09  KM411564 | Hap 12  KM411567 | Hap 13  KM411568 | Hap 14  KM411569 | Hap 15  KM411570 | Hap 16  KM411571 |
| *G. flavomarginatus* | 2 |  |  |  |  |  |  |  |  |  |  |  | 1 | 3 |  |
| *G. berlandieri* | 2 | 1 | 1 | 1 | 1 |  |  |  |  |  |  |  |  |  |  |
| *G. agassizii* - Western | 8 | 9 |  |  |  |  | 3 |  | 1 | 2 |  |  |  |  | 1 |
| *G. agassizii* - Northern | 6 | 5 |  |  |  | 4 | 2 |  | 1 |  |  |  |  |  |  |
| *G. agassizii* - Arizona | 10 | 14 |  |  |  | 1 |  |  | 1 | 4 |  |  |  |  |  |
| *G. morafkai* - Sonoran, Arizona | 21 | 37 |  |  |  |  |  | 5 |  |  |  |  |  |  |  |
| *G. morafkai* - Sonoran, Mexico | 9 | 16 |  |  |  |  |  | 2 |  |  |  |  |  |  |  |
| *G. morafkai* - Sonoran, ecotone | 6 | 10 |  |  |  |  |  | 1 |  |  |  | 1 |  |  |  |
| *G. morafkai* - Sinaloan, ecotone | 6 | 10 |  |  |  |  |  |  |  |  |  | 2 |  |  |  |
| *G. morafkai* - Sinaloan | 10 | 16 |  |  |  |  |  |  |  |  | 2 | 2 |  |  |  |
| Total | 80 | 118 | 1 | 1 | 1 | 5 | 5 | 8 | 3 | 6 | 2 | 5 | 1 | 3 | 1 |

| E. TB07 |  | Haplotype | | | | | | | | | | | | |
| --- | --- | --- | --- | --- | --- | --- | --- | --- | --- | --- | --- | --- | --- | --- |
| Taxa | *n* | Hap 01  KM411543 | Hap 02  KM411544 | Hap 03  KM411545 | Hap 04  KM411546 | Hap 05  KM411547 | Hap0 6  KM411548 | Hap 07  KM411549 | Hap0 8  KM411550 | Hap 09  KM411551 | Hap 10  KM411552 | Hap 11  KM411553 | Hap 12  KM411554 | Hap 13  KM411555 |
| *G. flavomarginatus* | 2 | 2 | 2 |  |  |  |  |  |  |  |  |  |  |  |
| *G. berlandieri* | 2 |  |  |  |  |  |  |  |  | 2 | 2 |  |  |  |
| *G. agassizii* - Western | 5 |  |  | 1 | 4 (3) |  |  |  | 2 |  |  |  |  | 3(3) |
| *G. agassizii* - Northern | 2 |  |  |  | 2(2) |  |  |  |  |  |  |  |  | 2(2) |
| *G. agassizii* - Arizona | 7 |  |  |  | 3(2) |  |  | 1 |  |  |  |  | 4 | 6(2) |
| *G. morafkai* - Sonoran, Arizona | 18 |  |  |  | 18(5) |  |  | 13 |  |  |  |  |  | 5(5) |
| *G. morafkai* - Sonoran, Mexico | 8 |  |  | 1 | 7 |  |  | 7 |  |  |  | 1 |  |  |
| *G. morafkai* - Sonoran, ecotone | 6 |  |  |  | 5(1) | 1(1) |  | 5(1) |  |  |  |  |  | 1(1) |
| *G. morafkai* - Sinaloan, ecotone | 6 |  |  |  | 4 | 2(1) | 1 | 5(1) |  |  |  |  |  |  |
| *G. morafkai* - Sinaloan | 10 |  |  |  | 4 | 6(3) | 3 | 7(3) |  |  |  |  |  |  |
| Total | 66 | 2 | 2 | 2 | 47 | 9 | 4 | 38 | 2 | 2 | 2 | 1 | 4 | 17 |

**Table S4**. **Overview of DNA polymorphism for mtDNA and 4 nDNA loci** estimated for tortoises in the genus *Gopherus*. bp = locus length in base pairs; Pop = putative population; S = number of polymorphic (segregating) sites; Hn = number of haplotypes; Hd = haplotype diversity; π = nucleotide diversity (per site); θ = theta (per site). Bold indicates significance value of *p* ≤ 0.05.

| Locus | bp | Pop | N | S | Hn | Hd | π | θ | Tajima's D |
| --- | --- | --- | --- | --- | --- | --- | --- | --- | --- |
| mtDNA | 1108 |  |  |  |  |  |  |  |  |
|  |  | *G. flavomarginatus* | 2 | 0 | 0 | 0 | 0 | 0 | - |
|  |  | *G. berlandieri* | 2 | 0 | 0 | 0 | 0 | 0 | - |
|  |  | *G. agassizii* - Mojave | 28 | 8 | 4 | 0.378 | 0.0021 | 0.0019 | 0.3732 |
|  |  | Goag- Northern | 6 | 2 | 3 | 0.733 | 0.0008 | 0.0008 | -0.0500 |
|  |  | Goag- Western | 22 | 0 | 0 | 0 | 0 | 0 | - |
|  |  | *G. morafkai* | 54 | 63 | 10 | 0.658 | 0.0211 | 0.0125 | **0.0125** |
|  |  | Gomo - Sonoran | 37 | 9 | 5 | 0.338 | 0.0006 | 0.0091 | -2.1008 |
|  |  | Gomo - Sinaloan | 17 | 6 | 5 | 0.640 | 0.0013 | 0.0016 | -0.5749 |
|  |  | desert tortoises | 82 | 89 | 14 | 0.782 | 0.0303 | 0.0163 | **2.8676** |
|  |  |  |  |  |  |  |  |  |  |
| BDNF | 640 |  |  |  |  |  |  |  |  |
|  |  | *G. flavomarginatus* | 4 | 0 | 1 | 0 | 0 | 0 | - |
|  |  | *G. berlandieri* | 4 | 1 | 2 | 0.500 | 0.0008 | 0.0009 | -0.6124 |
|  |  | *G. agassizii* - Mojave | 46 | 2 | 3 | 0.634 | 0.0012 | 0.0007 | 1.2103 |
|  |  | Goag- Northern | 8 | 1 | 2 | 0.250 | 0.0004 | 0.0004 | -1.0548 |
|  |  | Goag- Western | 38 | 2 | 3 | 0.661 | 0.0013 | 0.0007 | 1.4323 |
|  |  | *G. morafkai* | 104 | 2 | 3 | 0.414 | 0.0007 | 0.0006 | 0.2271 |
|  |  | Gomo - Sonoran | 72 | 2 | 3 | 0.393 | 0.0006 | 0.3930 | -0.0291 |
|  |  | Gomo - Sinaloan | 32 | 2 | 3 | 0.458 | 0.0008 | 0.0008 | 0.0055 |
|  |  | All desert tortoises | 150 | 4 | 5 | 0.575 | 0.0012 | 0.0011 | 0.0772 |
|  |  |  |  |  |  |  |  |  |  |
| R35 | 500 |  |  |  |  |  |  |  |  |
|  |  | *G. flavomarginatus* | 2 | 1 | 2 | 1 | 0.0020 | 0.0020 | - |
|  |  | *G. berlandieri* | 2 | 0 | 0 | 0 | 0 | 0 | - |
|  |  | *G. agassizii* - Mojave | 52 | 4 | 6 | 0.531 | 0.0026 | 0.0018 | 0.9952 |
|  |  | Goag- Northern | 12 | 4 | 4 | 0.788 | 0.0040 | 0.0027 | 1.7935 |
|  |  | Goag- Western | 40 | 4 | 5 | 0.318 | 0.0014 | 0.0019 | -0.6266 |
|  |  | *G. morafkai* | 102 | 7 | 8 | 0.709 | 0.0024 | 0.0027 | -0.2293 |
|  |  | Gomo - Sonoran | 72 | 6 | 7 | 0.619 | 0.0021 | 0.0025 | -0.3695 |
|  |  | Gomo - Sinaloan | 30 | 6 | 7 | 0.740 | 0.0027 | 0.0030 | -0.3111 |
|  |  | All desert tortoises | 154 | 7 | 9 | 0.783 | 0.0028 | 0.0025 | 0.2650 |
|  |  |  |  |  |  |  |  |  |  |
| TB02 | 425 |  |  |  |  |  |  |  |  |
|  |  | *G. flavomarginatus* | 4 | 1 | 2 | 0.500 | 0.0012 | 0.0013 | -0.6124 |
|  |  | *G. berlandieri* | 4 | 2 | 4 | 1.000 | 0.0031 | 0.0026 | 1.8931 |
|  |  | *G. agassizii* - Mojave | 48 | 4 | 6 | 0.631 | 0.0022 | 0.0021 | 0.0390 |
|  |  | Goag- Northern | 12 | 2 | 4 | 0.742 | 0.0023 | 0.0016 | 1.2896 |
|  |  | Goag- Western | 36 | 4 | 6 | 0.568 | 0.0020 | 0.0023 | -0.3495 |
|  |  | *G. morafkai* | 104 | 3 | 4 | 0.262 | 0.0006 | 0.0014 | -0.9331 |
|  |  | Gomo - Sonoran | 72 | 2 | 3 | 0.225 | 0.0005 | 0.0010 | -0.7345 |
|  |  | Gomo - Sinaloan | 32 | 2 | 3 | 0.331 | 0.0008 | 0.0012 | -0.6063 |
|  |  | All desert tortoises | 152 | 7 | 9 | 0.402 | 0.0012 | 0.0029 | -1.2941 |
|  |  |  |  |  |  |  |  |  |  |
| TB07 | 590 |  |  |  |  |  |  |  |  |
|  |  | *G. flavomarginatus* | 4 | 4 | 2 | 0.667 | 0.0045 | 0.0037 | 2.0803 |
|  |  | *G. berlandieri* | 4 | 3 | 2 | 0.667 | 0.0034 | 0.0028 | 2.0119 |
|  |  | *G. agassizii* - Mojave | 28 | 4 | 6 | 0.741 | 0.0034 | 0.0017 | 2.4879 |
|  |  | Goag- Northern | 4 | 4 | 2 | 0.667 | 0.0045 | 0.0037 | 2.0803 |
|  |  | Goag- Western | 24 | 4 | 6 | 0.768 | 0.0034 | 0.0018 | 2.3530 |
|  |  | *G. morafkai* | 96 | 5 | 7 | 0.687 | 0.0019 | 0.0017 | 0.3516 |
|  |  | Gomo - Sonoran | 64 | 5 | 6 | 0.628 | 0.0019 | 0.0018 | 0.1073 |
|  |  | Gomo - Sinaloan | 32 | 2 | 4 | 0.742 | 0.0017 | 0.0008 | 2.0368 |
|  |  | All desert tortoises | 124 | 5 | 9 | 0.742 | 0.0027 | 0.0016 | 1.5300 |
